# Integration of histopathological images and immunological analysis to predict M2 macrophage infiltration and prognosis in patients with serous ovarian cancer

**DOI:** 10.3389/fimmu.2025.1505509

**Published:** 2025-03-17

**Authors:** Ling Zhao, Jiajia Tan, Qiuyuan Su, Yan Kuang

**Affiliations:** ^1^ Department of Gynecology, First Affiliated Hospital of Guangxi Medical University, Nanning, China; ^2^ Department of Gynecology, Guangzhou First People’s Hospital, Guangzhou, China

**Keywords:** serous ovarian cancer, histopathological image features, ResNet18, M2 macrophage infiltration, deep learning artificial intelligence

## Abstract

**Objective:**

Investigating the effect of M2 macrophage infiltration on overall survival and to use histopathological imaging features (HIF) to predict M2 macrophage infiltration in patients with serous ovarian cancer (SOC) is important for improving prognostic accuracy, identifying new therapeutic targets, and advancing personalized treatment approaches.

**Methods:**

We downloaded data from 86 patients with SOC from The Cancer Genome Atlas (TCGA) and divided these patients into a training set and a validation set with a ratio of 8:2. In addition, tissue microarrays from 106 patients with SOC patients were included as an external validation set. HIF were recognized by deep multiple instance learning (MIL) to predict M2 macrophage infiltration via theResNet18 network in the training set. The final model was evaluated using the internal and external validation set.

**Results:**

Using data acquired from the TCGA database, we applied univariate Cox analysis and determined that higher levels of M2 macrophage infiltration were associated with a poor prognosis (hazard ratio [HR]=6.8; 95% CI [confidence interval]: 1.6–28, P=0.0083). External validation revealed that M2 macrophage infiltration was an independent risk factor for the prognosis of patients with SOC (HR=3.986; 95% CI: 2.436–6.522; P<0.001). Next, we constructed four MIL strategies (Mean probability, Top-10 Mean, Top-100 Mean, and Maximum probability) to identify histopathological images that could predict M2 macrophage infiltration. The Mean Probability Method was the most suitable and was used to generate a HIF model with an AUC, recall rate, precision and F1 score of 0.7500, 0.6932, 0.600, 0.600, and 0.600, respectively.

**Conclusions:**

Collectively, our findings indicated that M2 macrophage infiltration may increase prognostic prediction for SOC patients. Machine deep learning of pathological immunohistochemical images exhibited good potential for the direct prediction of M2 macrophage infiltration.

## 1 Introduction

Ovarian cancer (OC) is one of the most common gynecological cancers globally and the eighth leading cause of cancer-related death in women. OC accounted for approximately 3.7% of all cancer diagnoses and 4.7% of global deaths in 2020 (1). It is estimated that more than 150,000 OC-related deaths occur globally each year, and that approximately 12,740 of the US population will die from this malignancy by 2024 (2). Most cases (90%) of OC involve epithelial OC, a condition that can be divided into serous OC, mucinous OC, clear cell carcinoma, and endometrioid carcinoma; of these, the most common form is high-grade serous OC (SOC) (3). Due to the strong anatomical concealment of serous OC, most patients are diagnosed at an advanced stage of disease. The 5-year survival rate of patients with advanced OC remains low (4). Therefore, it is critical that we identify new prognostic characteristics for patients with SOC, improve individualized treatments, and promote the development of novel research.

A tumor is a highly complex system, composed predominantly of heterogeneous cancer cells, a variety of infiltrating immune cells, stromal cells, and a vascular structure, collectively referred to as a tumor microenvironment (TME), which can exert significantly effects on the occurrence, progression and metastasis of tumors and treatment responses (5). Macrophage polarization refers to the different functional states of macrophages in different immune microenvironments according to stimulation by different signals and cytokines. The most common polarization states are classical M1 and M2, which correspond to different immune responses and tissue repair processes, respectively. Typically, M1 macrophages are usually stimulated by pro-inflammatory cytokines (such as IFN-γ and TNF-α) and microbial products (such as LPS), which exert strong antibacterial and antiviral effects, and can promote the inflammatory response, mainly by producing a large amount of nitric oxide (NO), reactive oxygen species and cytokines (such as IL-1β and IL-6).

M2 macrophages are formed under the action of specific cytokines, such as IL-4 and IL-13, which are usually associated with tissue repair, and anti-inflammatory/immune regulation (6, 7). Some studies have shown that M2 macrophages support tumor growth and metastasis by secreting a variety of tumor-promoting factors, such as IL-10, TGF-β, and VEGF (8, 9). These cells not only promote angiogenesis and inhibit the immune response, but also enhance the invasiveness of tumor cells by remodeling the TME (10). In addition, M2 macrophages can destroy the basement membrane by secreting matrix metalloproteinases, thus helping tumor cells to invade the surrounding tissues (11). Recent studies have also shown that the polarization state of macrophages is not fixed, but has a high degree of plasticity and diversity. Even in different pathological environments, macrophages may exhibit a mixed phenotype of M1 and M2 types (10).

Based on the development of digital pathology and the advancement of computer algorithms, such as convolutional neural networks (CNN), fully convolutional networks, recurrent neural networks and generative adversarial networks, deep learning artificial intelligence is increasingly being applied in diagnostic disciplines based on image analysis, including pathology, ultrasound, radiology, ophthalmology and skin disease diagnosis (12). Following the introduction of full slide scanners in 1999, the application of artificial intelligence and computational methods in digital pathology has developed rapidly to digitally analyze full slide images. The creation of large-scale digital slide libraries, such as The Cancer Genome Atlas (TCGA), has promoted the substantive investigation of digital pathology and oncology by artificial intelligence (13). The ResNet18 network was first proposed by He et al. (14), which significantly improved the training effect and performance of deep networks by introducing a residual learning mechanism. The application of deep learning artificial intelligence in pathology helps us to overcome the limitations of subjective visual assessments by pathologists and integrate multiple measurements, including cells related to the tumor microenvironment (TME) for the precise treatment of tumors (15). Some studies have used machine learning to extract and identify pathological images for the diagnosis and classification of breast cancer (16). In another study, Javier et al. obtained automatic classification results for health, adenocarcinoma and squamous cell carcinoma based on the machine deep learning of lung histopathological images (17). These successful machine learning models provided a reference for us to introduce machine learning to investigate SOC. However, the integration of pathology into research involving the TME, which represents a nurturing ground for cancer, remains a largely uncharted domain. In this context, the focus on M2 macrophages, a specific subset of immune cells that represent a minor but significant fraction of the TME, is particularly lacking. These cells, despite their numerical minority, play an indispensable role in the immunosuppressive landscape of the TME and hold significant promise as therapeutic targets. To address this gap, we propose an approach that combines histopathological imaging for the analysis of M2 macrophages to enhance the predictive accuracy of prognosis for patients with SOC. This strategy promises to shed light on intricate dynamics within the TME and paves the way for more targeted and effective cancer therapies.

The purpose of this study was to investigate the effect of M2 macrophage polarization on the prognosis of patients with SOC, the prognostic value of histopathological image features (HIF), and the specific relationship between histopathology and M2 macrophage polarization. We analyzed data from the TCGA database and an external validation database, and demonstrated that M2 macrophage polarization represented an independent risk factor for SOC patients. In addition, the ResNet18 network was used to perform deep learning on the HIF to predict the level of M2 polarization in SOC patients. Finally, the prognostic performance was verified by internal and external validation sets to determine robustness and reliability.

## 2 Materials and methods

### 2.1 Sources and processing of data

**Figure 1:**
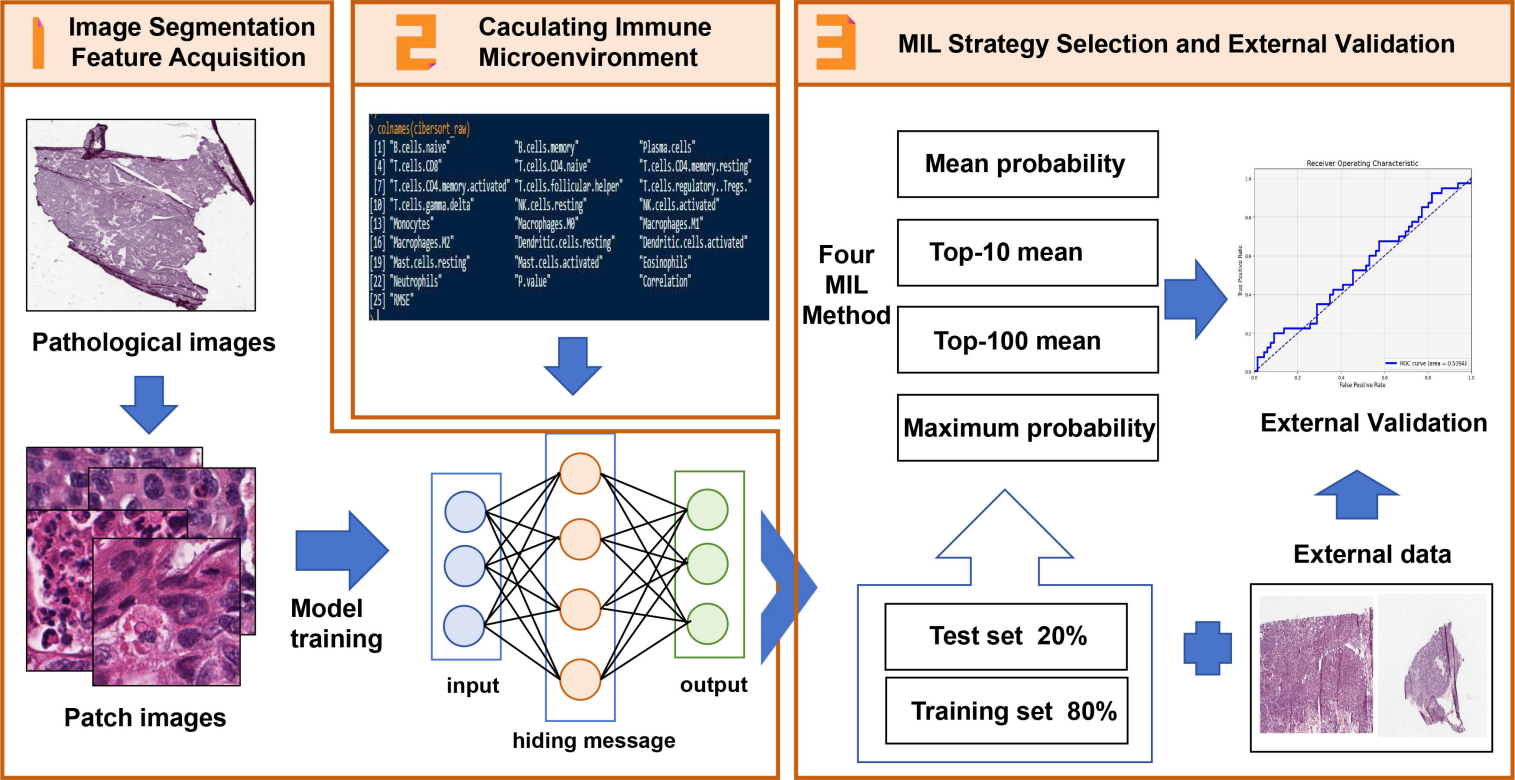
The Schematic of features extraction and construction of M2 macrophage infiltration prediction model. 1. The histopathological images of SOC patients were segmented into sub-images of 224x224 pixels and processed with ResNet18 to extract histopathological image features. 2. The M2 macrophage infiltration was calculated by CIBERSORT. The prognosis of patients with M2 macrophage infiltration was analyzed by univariate analysis, and M2 macrophage infiltration was predicted by image features. 3. By integrating the image features in the TCGA training set, a prediction model was established, and the independent risk factors and survival analysis of patients with M2 macrophage infiltration were analyzed by univariate and multivariate analysis. The TCGA test set and external data were used to evaluate its predictive value.

**Figure 1** depicts the processing of pathological images, the evaluation of infiltrating immune cells, and the establishment of models based upon the features of M2 macrophages. Data relating to cases from the TCGA data were divided into a modeling group and an internal verification group according to a ratio of 8:2. Step 2 used “CIBERSORT” (https://cibersort.stanford.edu/) to calculate the high and low infiltration of M2 macrophages. Step 1 used ResNet18 machine language to cut the Hematoxylin-Eosinstaining (HE) map from each case, identify characteristics of segmented images related to the high and low infiltration of M2, and perform repeated machine learning modeling. Step 3 involved internal and external data verification by identifying HE-segmented images to group cases with high and low infiltration of M2.

Formalin-fixed paraffin embedded (FFPE) sections are the gold standard for the diagnosis of diseases. Compared with frozen sections, FFPE sections are clearer and more suitable for computer analysis. FFPE slice images from 86 SOC patients in The Cancer Genome Atlas (TCGA, https://portal.gdc.cancer.gov/) were obtained from The Cancer Imaging Archive (TCIA, http://cancerimagingarchive.net/). We also downloaded corresponding clinical feature data, gene expression and tumor immune microenvironment data from TCGA. Finally, 106 SOC patients with histopathological images and immune infiltration were used to predict the features of M2 macrophages. In addition, we used an SOC tissue microarray and obtained associated clinical features from Shanghai Zhuoli Biotech Company (Shanghai, China). This tissue microarray included 106 SOC patients who underwent surgical and pathological diagnosis between April 2008 and September 2014. Follow-up ended upon the death of a patient or on the 30^th^ of July 2020. The study was approved by the ethics committee and received ethical approval (number: LLS M-15-01), and all patients provided informed consent.

### 2.2 Acquisition of histopathological imaging characteristics

Histopathological images in the TCGA database were in SVS format with extremely high resolution; consequently, these whole images could be used directly for feature acquisition. The ResNet18 model (Residual Network), a deep convolutional neural network widely used for image recognition tasks, was used to extract histopathological features from images. ResNet18 features 18 layers of depth, starting from a neural layer and four Res blocks. Each Res block contained two basic blocks and each basic block contained two convolutional layers and a final fully connected layer (1 + 4 × 4 + 1 = 18) (18) This was able to effectively extract important features from tissue images, including area, shape, intensity, granularity, texture and some complex measurement features, help pathologists make more accurate diagnoses and improve the efficiency of the diagnostic process. Key feature types of ResNet18 include: (1) Low-Level Features (Early Convolutional Layers) maninly contains (a) Layer 1 edge/texture primitive detection is to detect basic gradients, edges, and local textural patterns (e.g. nuclear membrane boundaries, cytoplasmic granularity). (b) Layer 2-3 intermediate textural complexity: captures co-occurrence of nuclei and stromal textures via larger receptive fields. Haralick-like features (contrast, entropy) emerge implicitly to quantify tumor-stroma interface irregularity. (2) High-level features (deep layers):Layer 4 or higher layer is to Identify morphological constellations: (a) Macrophage-specific patterns:”Clockface” chromatin patterns in macrophage nuclei and spatial clustering of small, round nuclei within fibrotic stroma. (b) Immune-stromal interaction features: lymphocyte exclusion zones around macrophage aggregates and collagen alignment adjacent to M2-rich regions.

### 2.3 Assessment of the immune microenvironment

Next, we used the CIBERSORT algorithm in R software 4.3.1 and the transcriptome profile of 86 patients with SOC in the TCGA database to calculate the proportion of different immune cells, especially the polarization and infiltration of M2 macrophages. The CIBERSORT algorithm (19) uses the principle of linear support vector regression to deconvolute the expression matrix of immune cell subtypes to estimate the abundance of immune cells. Using the calculated mean proportion of M2 macrophage infiltration as the boundary, we divided the 86 patients with SOC into a high M2 macrophage infiltration group and a low M2 macrophage infiltration group.

CD163 is a specific marker of M2 macrophages; therefore, using the external validation data, we performed immunohistochemistry on tissue microarrays prepared from 106 patients with SOC. Immunohistochemistry was performed by dewaxing in xylene at room temperature and hydration in a graded series of ethanol concentrations. Antigen retrieval was applied by microwave heating at 96°C to 98°C with antigen retrieval solution (sodium citrate buffer). Endogenous peroxidase in the tissue was inactivated by incubation with 3% hydrogen peroxide for 10 min. Tissue sections were incubated with blocking solution to block non-specific sites, and then incubated overnight at 4°C with anti-CD163 primary antibody (Abcam, ab182422). After washing with PBS, we added anti-rabbit IgG conjugated with horseradish peroxidase (HRP) and incubated at room temperature for 30 minutes. Finally, after washing with PBS, the sections were stained with 3’-diaminobenzidine (DAB) reagent. All sections were re-stained with hematoxylin, and sealed for microscopic examination after dehydration. The positive quantitative analysis of CD163 staining was performed by Image J software (Color Deconvolution), and mean values were calculated to divide the 106 patients with SOC into high and low infiltration groups.

### 2.4 Statistical analysis

#### 2.4.1 Data preprocessing

First, we attempted to use immunohistochemical image features to predict M2 macrophage infiltration. After matching the calculated M2 macrophage infiltration group with immunohistochemical images, we excluded 21 SOC patients without M2 macrophage infiltration and included the remaining 65 SOC patients in our final analysis. First, we set the environment, checked the need to use CUDA (Compute Unified Device Architecture), and gave priority to the use of GPU (Central Processing Unit) to accelerate training. This is a common practice in deep learning, because GPU is more efficient than CPU in processing parallel operations. Next, we used the Python “HE_Patches_Dataset” to create the dataset, and the “torch.utils.data.Dataset” to process the image file, from which the path and label of the image were read from the CSV file. All patch images were uniformly scaled to 224 × 224 pixels, converted to Tensor format, and normalized to match the input requirements of the pre-trained ResNet18 model. Next, we used “train_test_split” in Python to divide the dataset into a training set and a validation set, with a ratio of 80% training set and 20% validation set. Then, “DataLoader” in Python was used for data batch processing, and “shuffle=True” was applied to the training set to disrupt the data, which was helpful for model learning.

#### 2.4.2 Model construction

Next, the model was initialized, and the pre-trained ResNet18 model was applied. The pre-trained model accelerated the training process and improved model performance. The output feature number of the last fully connected layer was modified to two to adapt to the binary classification task. Further training settings included the “cross entropy loss function” for classification problems. Using the Stochastic Gradient Descent (SGD) optimizer with momentum, the learning rate was set to 0.001 and the momentum was 0.9. Momentum helped to accelerate the convergence of SGD in the relevant direction and suppress oscillations. The model was trained for 25 epochs. At each epoch, we calculated the cumulative loss and accuracy, and the model was evaluated with the validation set. After each epoch, the best model was saved by comparing accuracy with the validation set, which prevented model overfitting. The final model was saved only when the highest accuracy was achieved with the validation set.

#### 2.4.3 Evaluation and optimization of the model

The accuracy, recall rate, precision, F1 score and Area Under the Receiver Operating Characteristic Curve (AUC-ROC) values were used to evaluate the performance of the model, and accuracy was used as the main index. In the training process, the accuracy of the validation set was continuously compared to ensure the generalization ability of the model on the unseen data. Grad-CAM (20) was used to generate a heat map to visualize the area of interest in the model. In multiple instance learning (MIL), the diagnosis of patients was usually based on information extracted from multiple tissue samples or a “patch.” Here, each patient could be considered as a “bag,” and each patch was an “instance” in the bag. Based on the prediction of these examples, we used four methods (Mean Probability, Top-10 Mean, Top-100 Mean and Maximum Probability) for preliminary evaluation, and then selected the best strategy to determine the overall prediction results for each patient.

## 3 Results

### 3.1 Determination of immune infiltration

**Figure 2:**
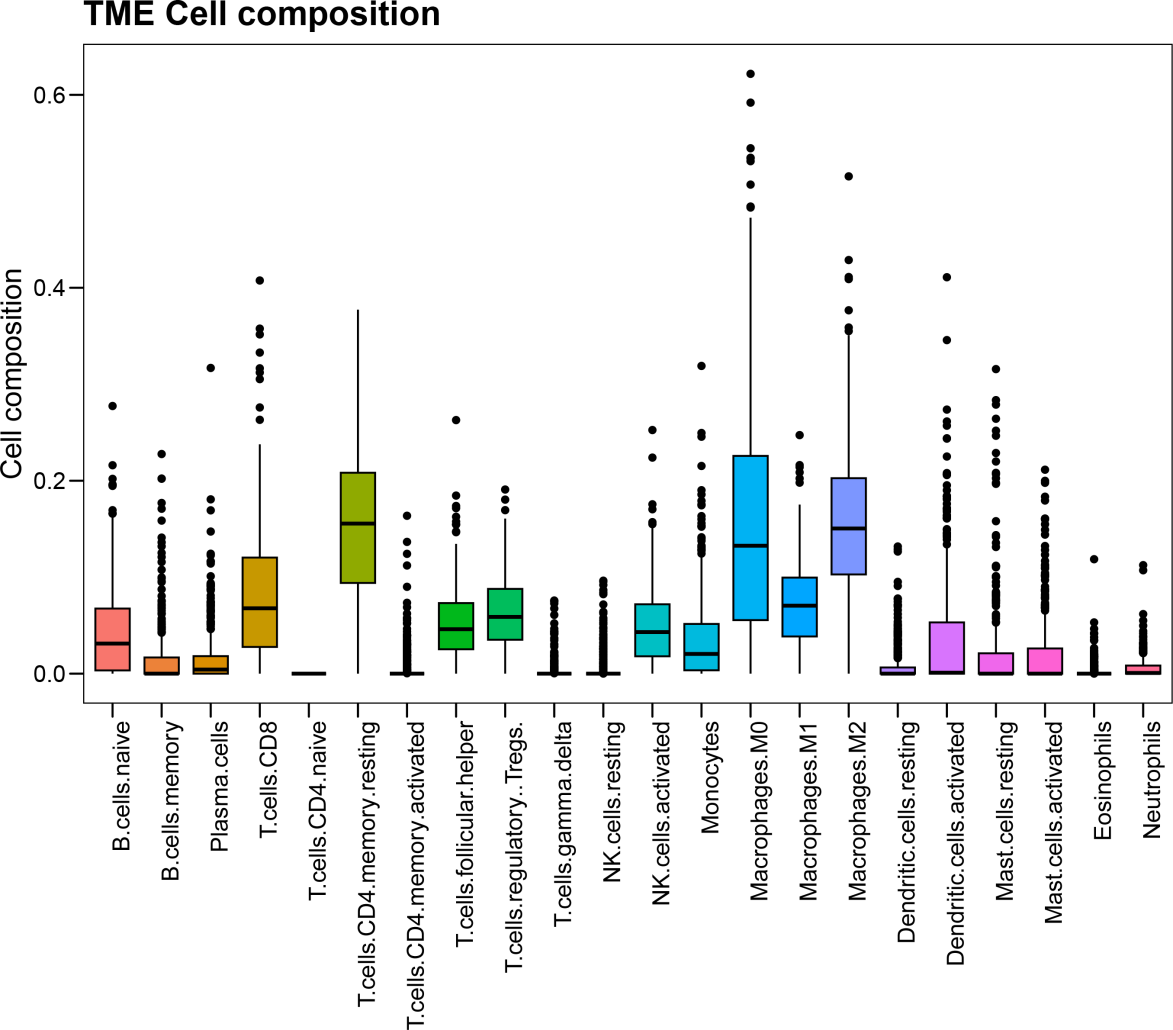
The composition and corresponding proportion of immune microenvironment in SOC patients in TCGA database.

First, we used the CIBERSORT algorithm to calculate immune cell infiltration in SOC patients extracted from the TCGA database (**Supplementary Table 1**). The immune cell types included native B cells, memory B cells, plasma cells, CD8 T cells, naïve CD4 T cells, memory resting CD4 cells, CD4 memory activated T cells, follicular helper T cells, regulatory Tregs T cells, gamma delta T cells, resting NK cells, activated NK cells, monocytes, M0 macrophages, M1 macrophages, M2 macrophages, resting dendritic cells, activated dendritic cells, resting mast cells, activated mast cells, eosinophils and neutrophils. The overall composition of immune cell infiltration is shown in **Figure 2**. Compared to other cells, the proportion of CD4 memory resting T cells was the highest, followed by M2 macrophages. Univariate Cox regression analysis was used to evaluate the effect of macrophages M1 and M2 on the overall prognosis of patients with SOC. Analysis revealed that a high proportion of M1 macrophages was associated with improved prognosis (HR=0.018, 95% CI: 0.0014–0.25, P=0.0028) and high level of M2 macrophages was related to poor prognosis (hazard ratio [HR]=6.8; 95% CI [confidence interval]: 1.6-28; P=0.0083).

### 3.2 Deep learning modeling of immunohistochemical images

**Figure 3:**
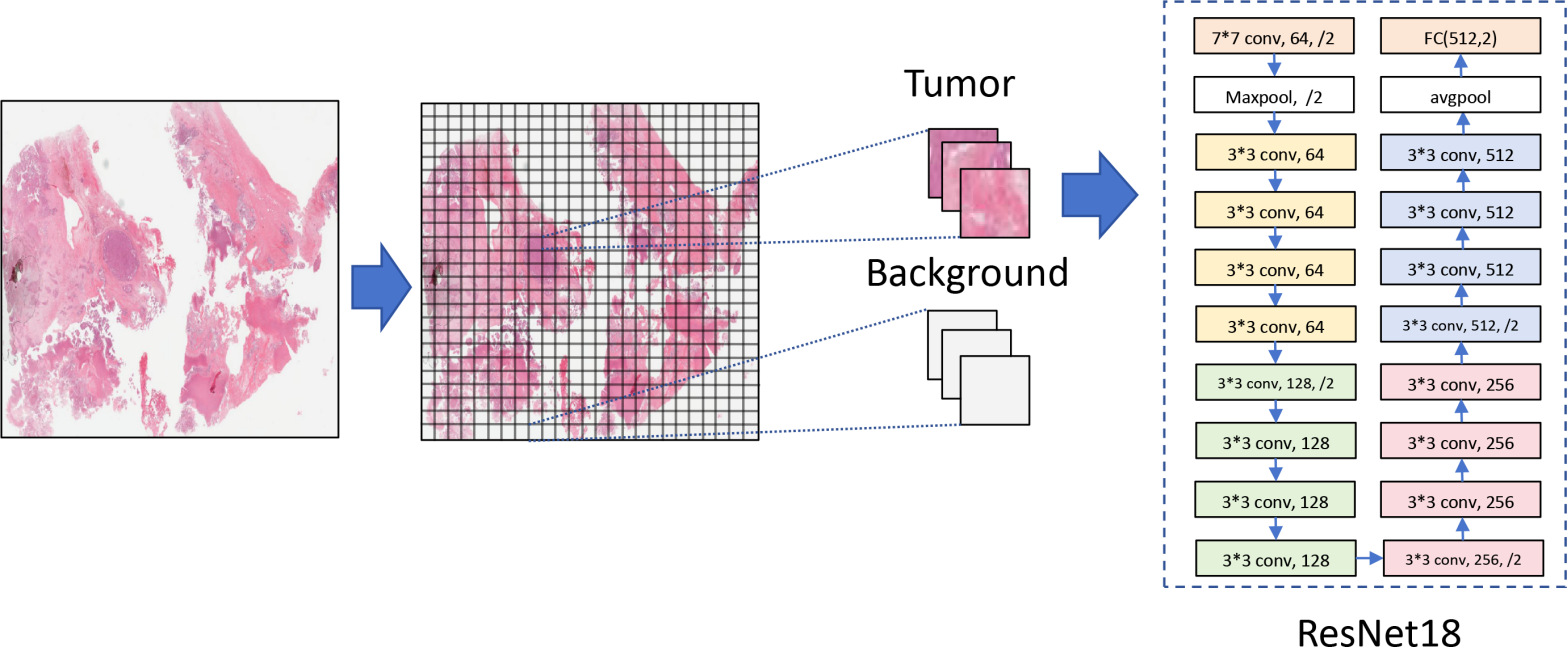
Background reduction and features extraction from patches.

Next, we attempted to directly detect the histopathological features of SOC by using CNN to predict M2 macrophage infiltration, and downloaded scanned images of tumor pathology from TCGA for further data preprocessing. These tissue pathological sections were cut into small images of 224 × 224 pixels, and each image was standardized using specific means (0.485, 0.456, 0.406) and standard deviations (0.229, 0.224, 0.225) to match the input format of ResNet18. In order to ensure that each patch had a complete organizational structure and reduce the proportion of background, we calculated the color value of each patch. Since the background was white, the lower the color value of the patch map, the greater the background. Therefore, we set the color threshold to reduce the background map of 95% patches, and extract the next feature of the remaining patch (**Figure 3**). Therefore, the acquired images were used for in-depth learning and to train CNN. Then, we divided the SOC patients into an 80% training set and a 20% validation set, and used the last three blocks (18 layers) of ResNet18 to train the images.

### 3.3 Accuracy and robustness of the model

**Figure 4:**
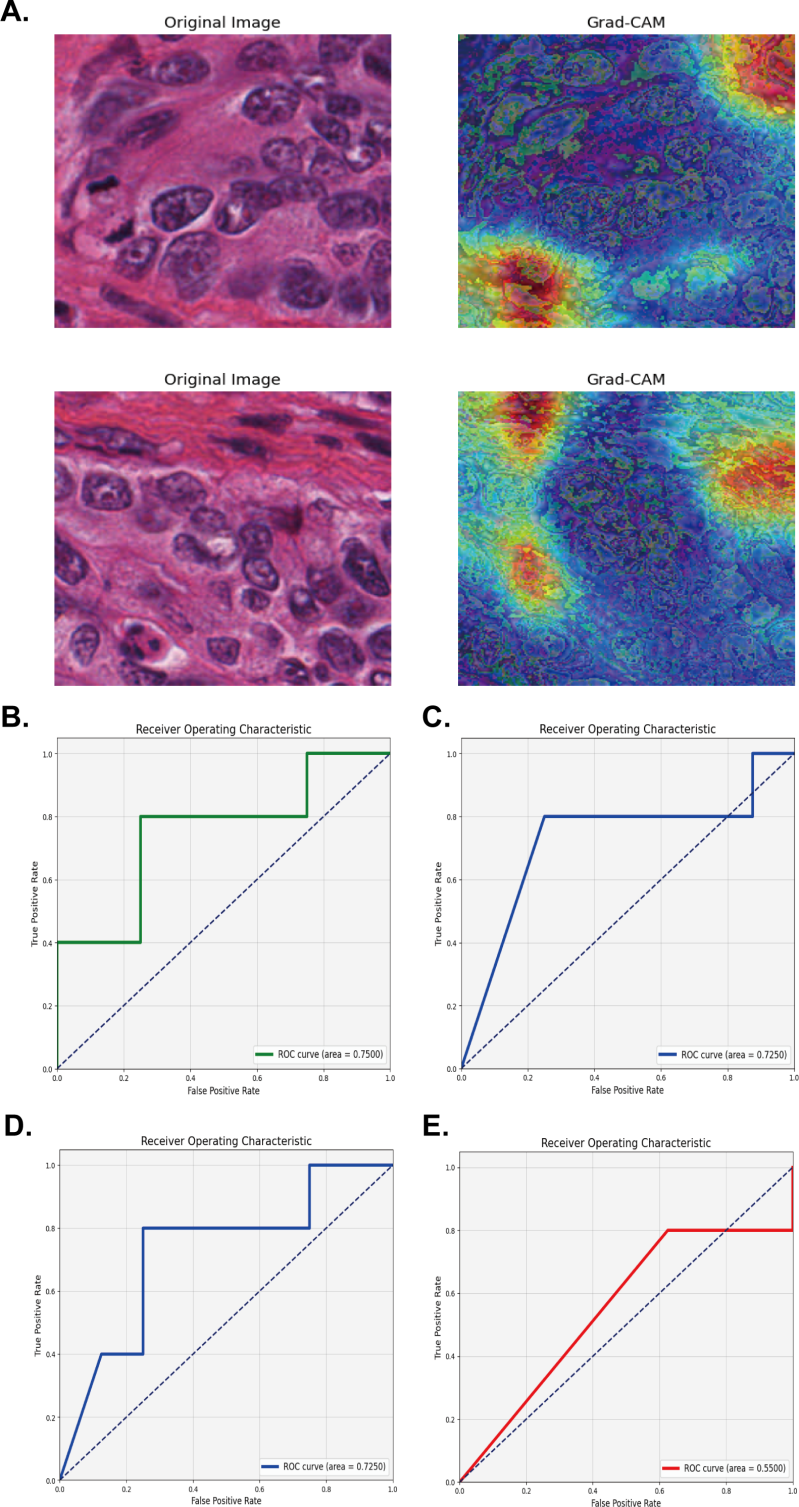
Validation of the model using four multiple instance learning strategies for M2 macrophage infiltration prediction. **(A)** Segmented HE Staining and Grad-CAM Images for extracting and predicting M2 macrophage infiltration. **(B-E)** The AUC area with Mean probability, Top-10 mean, Top-100 mean, and Maximum probability strategy in the TCGA test set.

Grad-CAM was able to accurately identify and highlight which regions of input images were important for the prediction of high and low M2 macrophage infiltration for the network. This method utilized the activation of the last convolutional layer to generate class activation mapping, retained the architecture of the deep model, and provided visual interpretation without affecting accuracy. The left side of **Figure 4A** shows the HE staining images after segmentation, while the right side shows the Grad-CAM images, in which the red and yellow areas of the HE image were important for the prediction of the network’s classification of M2 polarization infiltration. Grad-CAM decoded the importance of each feature map for the classification of M2 polarization infiltration by analyzing the gradient in the last convolution layer.

For pathological application, four MIL methods (Mean Probability, Top-10 Mean, Top-100 Mean, and Maximum Probability) were used to predict a patient’s disease status based on the features extracted from multiple pathological sections. For example, we were able to classify each image based on specific features (such as cell morphology and tissue structure) extracted from each image, and used one of the MIL strategies to synthesize this information, and finally obtain an overall diagnosis of the patient. The Mean Probability Method assumes that each instance in the bag is equally important, and the final prediction of the bag is determined by calculating the mean of the prediction probabilities for all instances in the bag. When we used the Mean Probability strategy, the histopathological image features (HIF) model exhibited a good AUC of 0.7500; accuracy, recall rate, precision and F1 score were 0.6932, 0.600, 0.600, and 0.600, respectively (**Figure 4B**). The Top-10 Mean Method only considered the predictions of the 10 most likely instances in the bag and averaged these probabilities. This method is more diagnostically valuable for some scenarios (such as abnormal tissue samples) than for other scenarios. In this study, the Top-10 Mean was also used for the HIF model with an AUC, accuracy, recall rate, precision and F1 score of 0.7250, 0.3846, 1.000, 0.3846, and 0.5556, respectively (**Figure 4C**). Similar to the Top-10 Mean model, the Top-100 Mean method considered more instances (100) when selecting instances for averaging, which may be more appropriate with a large number of instances. Using the Top-100 Mean Method for the HIF model, the AUC, accuracy, recall rate, precision, and F1 score of the HIF model were 0.7250, 0.4615, 0.8000, 0.4000, and 0.5333, respectively (**Figure 4D**). The Max Probability Method takes the prediction of the instance with the highest probability in the bag as the prediction for the whole bag. When employing the Max Probability Method for the HIF model, the AUC, accuracy, recall rate, precision and F1 score were 0.5500, 0.3846, 1.0000, 0.3846, and 0.5556, respectively (**Figure 4E**). Therefore, after comprehensive evaluation, we chose the Mean Probability Method to apply the MIL as this was more suitable for our specific samples.

### 3.4 External validation of the model

**Figure 5:**
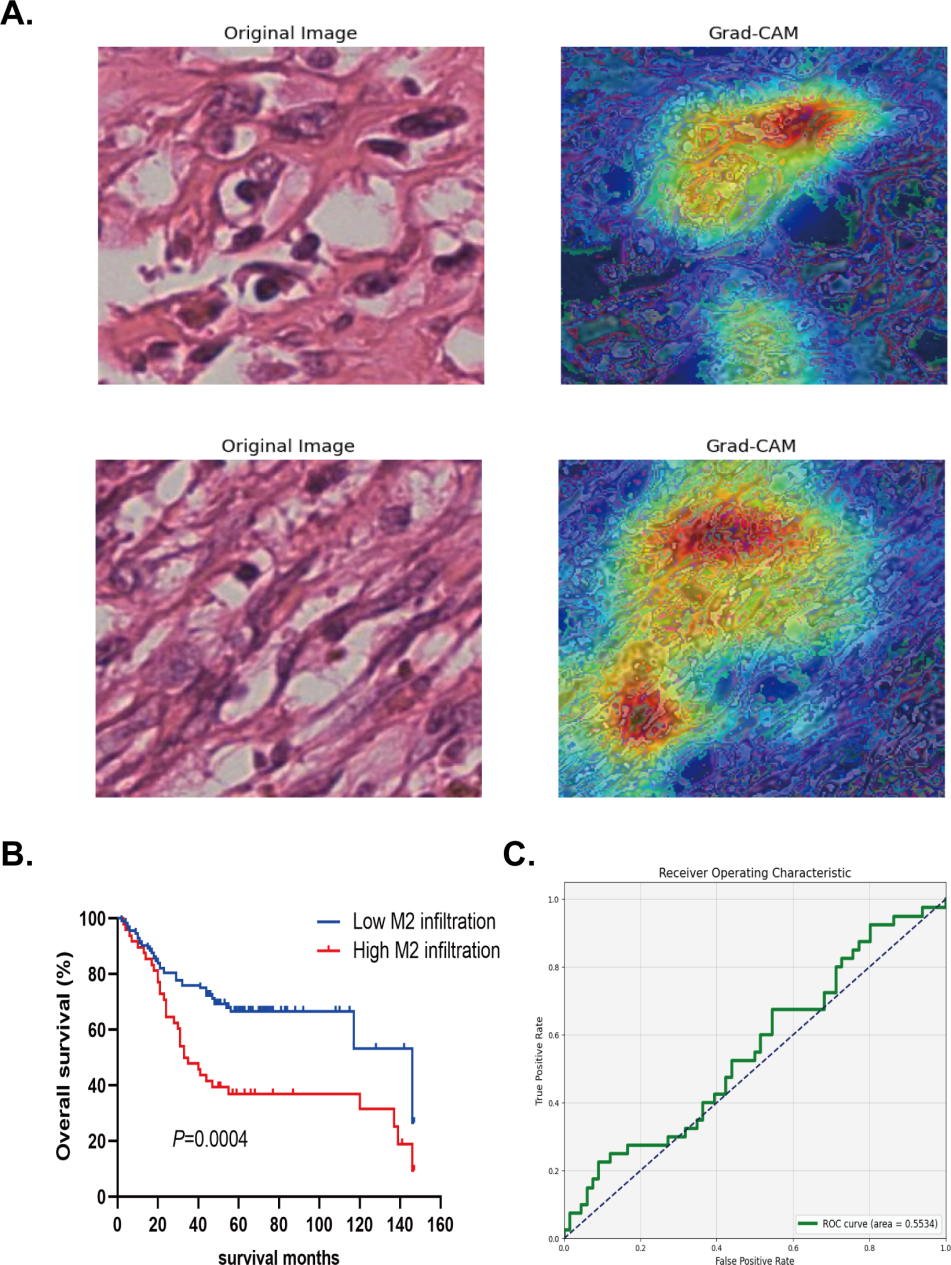
The verification of deep machine learning model in external data. **(A)** Segmented HE Staining and Grad-CAM Images for predicting M2 macrophage infiltration. **(B)** Kaplan-Meier method was used to analyze the survival analysis of high and low infiltration groups of M2 macrophage in the external data. **(C)** The AUC area with Mean probability method in the external data.

**Figure 6:**
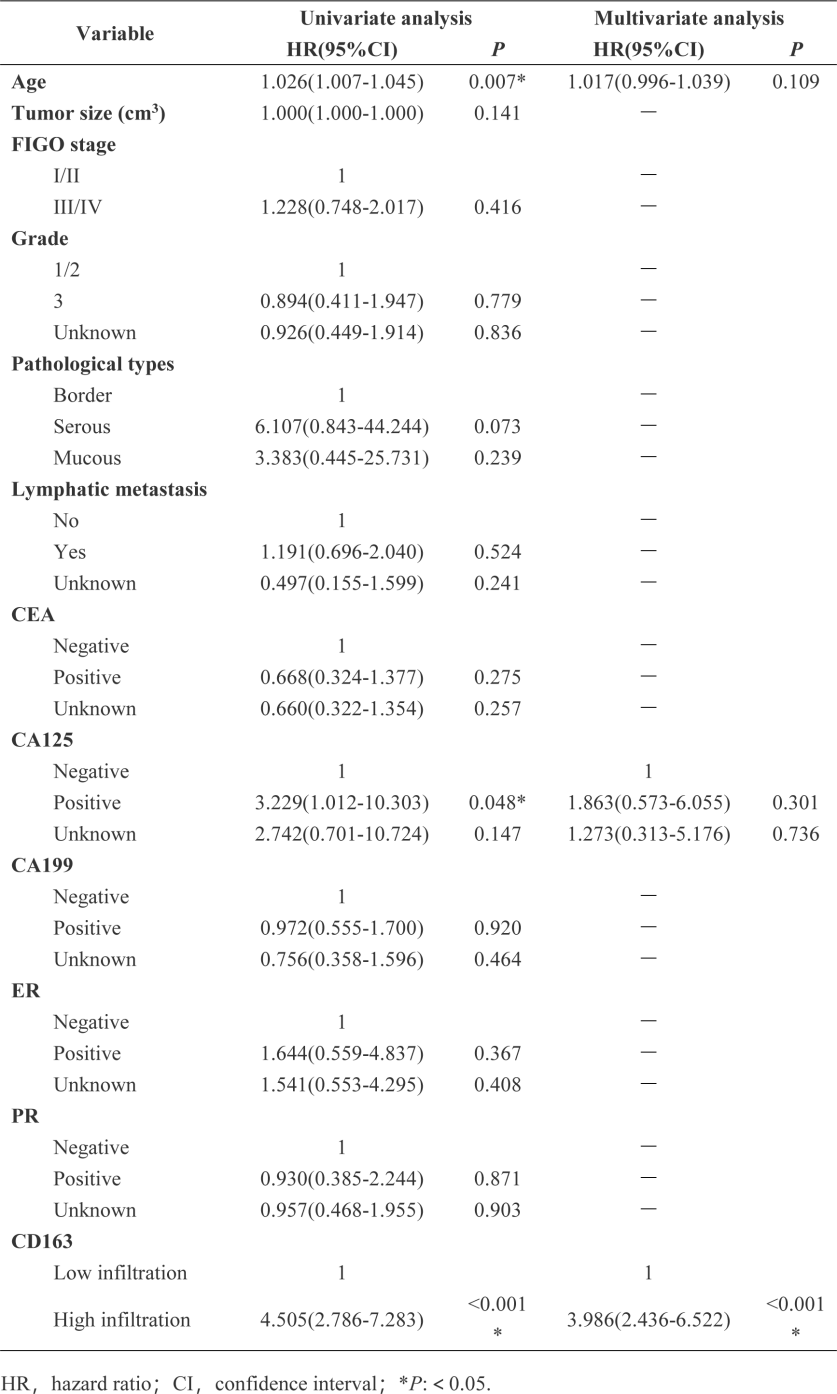
Univariate and multivariate COX analysis of the relationship between clinical characteristics and overall survival was performed in external validation data.

The left side of **Figure 5A** shows the HE staining image after cutting in the verification set, while the right side shows the corresponding Grad-CAM image. By performing clinical survival analysis, we found that the survival of patients with a high infiltration level of M2 macrophages was poor (**Figure 5B**). Univariate and multivariate Cox analysis also demonstrated that M2 macrophage infiltration was an independent risk factor affecting the prognosis of patients with OC (HR=3.986; 95% CI: 2.436–6.522; P<0.001) (see **Figure 6** for further details). When applying the Mean Probability Method, the corresponding AUC was 0.5534 (**Figure 5C**).

## 4 Discussion

In the present study, we found that the level of M2 macrophage polarization was an independent risk factor for patients in SOC patients, as determined by univariate and multivariate Cox analysis. Next, we used the ResNet18 network to learn pathological images from SOC patients, and extracted a series of important image features. These image features were then used by machine learning to identify the level of M2 macrophage polarization in tumors. Finally, we constructed a model to determine the level of M2 polarization with image feature prediction by machine training, and used internal and external validation groups to verify the predictive effect of the model, further demonstrating that the constructed model had good accuracy and robustness.

The existence of M2 macrophages is closely related to the malignant characteristics of SOC (21). M2 macrophages have been shown to enhance tumor angiogenesis and immune escape mechanisms by secreting pro-tumor factors, which are considered to be key factors for increased cancer invasiveness and a poor prognosis (10, 22, 23). Emerging pan-cancer studies highlight mechanisms that reinforce the prognostic significance of M2 macrophages in SOC. EPHB2, identified as a predictive biomarker for immunotherapy response and survival across cancers (24), may modulate M2 macrophage polarization, linking their immunosuppressive functions to therapeutic resistance. Similarly, strategies targeting T cell exhaustion (25) underscore the role of M2 macrophages in fostering immune evasion. LMNB2, a diagnostic and prognostic biomarker in lung cancer (26), correlates with genomic instability and proliferation potentially amplified by M2-derived cytokines. Furthermore, multi-omics analyses of malignant cell-associated ligand–receptor networks (27) reveal crosstalk between tumor cells and stromal components, suggesting M2 macrophages may drive SOC progression through similar paracrine signaling. By transcending traditional markers, this integrative approach highlights novel therapeutic targets and reinforces M2 macrophages as pivotal prognostic determinants. In this study, both the internal data set and the external data set proved that the polarization level of M2 macrophages was associated with the prognosis of SOC patients; the higher the level, the worse the prognosis. Although the findings of previous studies were consistent with the results generated by our study, few studies have verified this method as an independent prognostic indicator by analyzing large-scale databases and applying efficient image analysis techniques. Our study not only verified the role of M2 macrophages in SOC, but also provided a quantitative basis for the prognostic evaluation of M2 macrophages for the first time by applying pathological image analysis facilitated by artificial intelligence.

The gold standard for the diagnosis and treatment of cancer patients is based on the pathological diagnosis of tissues. Histopathological images contain information relating to the morphological characteristics of tumor cells and their microenvironment, and may represent important biomarkers for the survival outcomes of cancer patients (28). However, at present, pathological examination reports are diagnosed by multiple pathologists based on their experience, and this type of subjective evaluation often ignores the large quantity of information provided by pathological sections. With the continual improvement of computer algorithms, deep learning machines have been developed to assist feature extraction from images of pathological sections, and these features have been shown to be related to the prognosis of tumors (29–31). Additionally, the automatic extraction of image features by a machine not only improves efficiency and reduces costs, but also reduces the occurrence of misdiagnosis and missed diagnosis. Notably, cutting-edge advances underscore the transformative potential of converging machine learning with multi-omics analytics. Ye et al. engineered the iMLGAM framework that synergistically integrates genetic algorithms and machine learning to decode tumor microenvironment dynamics from multi-omics data, yielding critical insights for predicting immunotherapy responses across malignancies (32). Machine deep learning has also been used to investigate the pathology of SOC. In a previous study, Boehm et al. collected multimodal data sets from 444 patients with primary advanced high-grade SOC, and performed risk stratification for patients by integrating histopathological, radiological and clinical genomics machine learning models (33). In another study, Zeng et al. established models for BRCA1 mutation, BRCA2 mutation, high microsatellite instability, microsatellite stability, and different molecular subtypes (proliferative, differentiated, immunoreactive, and interstitial) by performing machine learning on pathological tissue images held by the TCGA database (34). These previous studies mainly focused on the pathological features of SOC, including tissue structure, tumor grade, and molecular subtype. However, few deep learning studies have focused on immune cells in combination with pathology.

Second, previous studies (35, 36) have focused on the role of M2 macrophages in SOC and their relationship with the TME and patient prognosis. The existing literature generally utilized immunohistochemistry, flow cytometry, and other methods to quantitatively analyze macrophage infiltration. Zheng et al. (37) used single cell analysis to analyze the significance of the density, spatial distribution and gene expression of tumor-associated macrophage phenotypes as a prognostic factor for the overall survival of patients with lung cancer. However, most of these studies relied on traditional immunological analysis methods, and there was a clear lack of research on the integration of HIF and immunological data. In terms of model selection, the present study utilized four different MIL strategies to process histopathological images. Compared with the traditional method used to analyze single image features, the MIL method can effectively process image data containing complex information, and then select and fuse features through different strategies, thus improving the generalization ability and predictive performance of the model. Although previous studies have mostly utilized deep learning networks, such as CNN (12, 38) for image classification, the MIL strategy applied in this study was able to better capture the distribution pattern of M2 macrophages in the TME by integrating multiple local instance information, thus providing a new method for the precision clinical management of OC.

Although this study has achieved important results, there are also some limitations that need to be considered. First, the limited sample size may have influenced the universality of our findings. The two cohorts in this study provided a relatively small cohort, and may have influenced the performance of the model. Notably, in the verification queue, the accuracy and stability of the machine learning model were limited. The small sample size may have led to a higher degree data fitting to the model, thus affecting its generalization ability in new and untested data. This phenomenon was particularly significant in the validation cohort, which may manifest manifested as the overfitting of the model to specific data features, resulting in a decline in predictive performance. On the contrary, the validation data for M2 polarization quantification was derived from immunohistochemistry, which may have differed from the sequencing data in modeling. Second, this study mainly focused on the number and distribution of M2 macrophages, but did not investigate the specific functional mechanism involved. For example, we still need to investigate how M2 macrophages might affect the biological behavior of tumors through specific signaling pathways. This type of research will help to reveal the specific role of M2 macrophages in the TME, thus providing a theoretical basis for the development of targeted therapy. Moreover, while we utilized HIF to elucidate the role of M2 macrophages within the TME, it is crucial to acknowledge the complexity of the TME, a system that is influenced by a multitude of factors that extend well beyond the scope of M2 macrophages alone. The TME comprises a dynamic interplay of various cell types, signaling molecules, and metabolic pathways, all of which contribute to the heterogeneity and complexity of the tumor niche. Therefore, it is imperative for future research to place a strong emphasis on the comprehensive diversity of the TME. Such an approach is essential if we are to gain a more comprehensive understanding of how the TME influences tumor progression and may lead to the identification of novel therapeutic targets. Finally, The external dataset (TCGA) differs from our internal cohort in demographic and clinical characteristics, such as age distribution, cancer stage, and treatment protocols. These discrepancies may introduce unmeasured confounding effects, altering risk associations. To improve clinical applicability, we propose that future iterations of the model incorporate treatment-related covariates and validate it in prospectively annotated, multi-institutional cohorts with standardized therapeutic protocols. However, despite this limitation, our current findings still have important clinical and scientific value. The integration of histopathological images and immunological analysis provided a new concept to comprehensively investigate the immune characteristics of the TME, especially the role of M2 macrophages. This method not only provides strong support for the prognostic prediction of SOC, but also provides a reference for the analysis of immunological characteristics of other tumor types. Although the small sample size may have limited the generalization of the model, the collection and analysis of high-quality data ensured the reliability and rigor of our results. Our findings provide a solid foundation for future research; once the sample size has been expanded, the accuracy and stability of the model may be further improved.

## Conclusion

In this study, we demonstrated that the level of M2 macrophages was an independent risk factor for the prognosis of patients with SOC. In addition, we confirmed the potential ability of features extracted from histopathological images to predict the polarization level of M2 macrophages in patients with SOC. Our findings are expected to help pathologists and clinicians to evaluate the prognosis of SOC patients and provide valuable reference for individualized treatment.

## Data Availability

The raw data supporting the conclusions of this article will be made available by the authors, without undue reservation.

## Supplementary material

Supplementary Table 1 The immune cell infiltration of SOC patients in the TCGA database.
